# Meta-analysis of the effects of exercise intervention on physical health in individuals undergoing compulsory isolation

**DOI:** 10.3389/fpsyt.2026.1748610

**Published:** 2026-05-01

**Authors:** Zhiyao Zhang, Zhuo Zhou, Hongmei Sun, Qinglu Wang, Maolin Zhang

**Affiliations:** 1 Graduate School of Shandong Sport University, Jinan, Shandong, China; 2 College of Sports and Health, Shandong Sport University, Jinan, Shandong, China; 3 Wushu School of Shandong Sport University, Jinan, Shandong, China

**Keywords:** aerobic training, balance, drug dependence, forced abstinence, pulmonary function, reaction time, traditional Chinese exercise

## Abstract

**Background:**

Physical health is the basic indicator to evaluate the health of drug addicts after the process of drug rehabilitation. In order to better improve the deficiency degree of physical health of drug addicts, it is necessary to carry out a systematic review.

**Objective:**

To explore the effects of exercise intervention on the physical health of individuals undergoing compulsory drug rehabilitation using Meta-Analysis, aiming to provide evidence-based support for improving their physical health.

**Methods:**

Randomized controlled trials (RCTs) published between 2019 and December 2024, examining the impact of exercise intervention on the physical health of compulsory detoxification individuals, were retrieved from databases including Web of Science, PubMed, Cochrane Library, Medline, China National Knowledge Infrastructure (CNKI), Wanfang Data, and VIP Chinese Journal Database. The quality of included studies was assessed using the Cochrane risk-of-bias assessment tool. RevMan 5.4 software was employed for heterogeneity testing, effect size synthesis (using mean difference [MD] and 95% confidence interval [CI]), and generation of forest plots, funnel plots, and quality assessment diagrams. Subgroup analyses were performed to evaluate sensitivity and heterogeneity of the included studies.

**Results:**

Exercise intervention effectively improved the physical health of compulsory drug rehabilitation individuals, particularly in physical fitness indicators: sit-and-reach test [MD = 3.92, 95%CI = (3.23, 4.62), P<0.001], single-leg standing with eyes closed [MD = 7.03, 95%CI = (6.05, 8.02), P<0.001], grip strength [MD = 1.23, 95%CI=(0.06, 2.39), P = 0.04], and choice reaction time [MD=-0.03, 95%CI=(-0.05, -0.01), P = 0.002]. Improvements in physical function were also observed; however, the increase in vital capacity [MD = 86.81, 95%CI=(-1.56, 175.17), P = 0.05] did not reach statistical significance.

**Conclusion:**

This meta-analysis provides evidence that exercise intervention significantly improves specific physical health deficits—namely flexibility (sit-and-reach), balance (single-leg stance), muscular strength (grip strength), cardiopulmonary function (vital capacity), and sensorimotor coordination (choice reaction time)—in individuals undergoing compulsory rehabilitation. It is recommended to adopt a combination of aerobic and traditional fitness exercises, with at least 3 sessions per week, each lasting no less than 40 minutes, and a duration of over 12 weeks, providing scientific evidence for drug rehabilitation practices. These indicators were selected because they directly reflect the multisystem damage (muscular, neural, and cardiorespiratory) caused by chronic substance use. However, this study acknowledges the limitation that psychological and neurocognitive outcomes (e.g., cravings, mood, executive function), which are crucial in addiction treatment, were not included in the eligibility criteria and systematic analysis. The follow-up research will combine physical and psychological indicators to conduct a comprehensive evaluation of the intervention effect of exercise on drug rehabilitation.

**Systematic review registration:**

https://www.crd.york.ac.uk/prospero/, identifier CRD420251029820.

## Background

Globally, the drug abuse constitute a major public health threat to human health and social stability (1). According to the 2024 China Drug Situation Report (2) The number of drug addicts across the country continues to rise, causing severe damage to individuals’ physical and mental health (3, 4). Meanwhile, drug addiction, as a chronic relapsing disease (5), damages the nervous, immune and cardiovascular systems, disrupts neuromuscular coordination, and impairs physical fitness (e.g., upper-limb strength, flexibility, balance) and reaction capacity, severely diminishing quality of life and physical health (6). Chronic drug use, particularly methamphetamine and opioids, induces profound physiological damage to multiple body systems. At the neuromuscular level, drugs of abuse disrupt protein synthesis pathways, accelerate muscle fiber degradation, and impair mitochondrial function, leading to muscle atrophy and reduced strength; The nervous system sustains equally severe damage: neurotoxicity affects dopaminergic and serotonergic pathways, damages motor neurons, and impairs neuromuscular junction transmission, resulting in diminished motor coordination, prolonged reaction times, and balance deficits (6). Consequently, improving the physical health of individuals undergoing compulsory rehabilitation remains a critical focus and research hotspot in drug rehabilitation work.

Currently, traditional rehabilitation methods exhibit limitations in addressing the complexity of drug addiction, so it is extremely urgent to explore more effective auxiliary means for drug rehabilitation. Although various treatment methods for drug dependence have been developed, including pharmaceutical, psychological, and sociological interventions, their intervention effects on physical health are often insignificant (3). Pharmaceutical interventions alleviate physical discomfort but fail to directly improve the organic damage in individuals undergoing compulsory rehabilitation, including their physical health. Psychological interventions aim to improve mental states, yet their impact on physical fitness must be achieved through a chain of active exercise and dietary improvements, which entails a certain degree of dependency and indirectness. Sociological strategies target relapse risk and social reintegration but exert minimal impact on physical health due to external environmental constraints.

Exercise intervention, a well-established non-pharmacological approach with decades of scientific validation, has gradually attracted widespread attention, demonstrating unique potential in improving the physical health of drug addicts (7). It can exert positive effects through exercise intervention. Additionally, the physical health of individuals undergoing compulsory rehabilitation mainly encompasses body morphology, physical fitness, and physical functions. Studies have confirmed that exercise intervention not only improves the physical fitness of these individuals but also alleviates issues related to their physical functions (8). Twelve weeks of aerobic exercise can effectively improve the physical fitness of drug users and enhance their cardiopulmonary capacity (9, 10). However, some studies have found that the effect of exercise intervention on the physical health of individuals undergoing compulsory rehabilitation is not significant. A 12-week randomized controlled trial showed that Tai Chi exercise had no positive impact on BMI(body mass index), with no statistically significant difference between the results before and after the intervention. Nevertheless, changes in BMI may depend on the frequency and intensity of exercise (4).

Therefore, the effects of exercise interventions with varying kinematic characteristics on the physical health of individuals undergoing compulsory rehabilitation warrant further in-depth exploration and research analysis. Consequently, this study employs a meta-analysis approach to systematically synthesize existing kinematic indicators related to the physical health of compulsory detoxification individuals in exercise interventions, with sit-and-reach, choice reaction time, single-leg stance with eyes closed, grip strength, and vital capacity as the outcome measures. It will compare and analyze the improvement effects of different exercise modalities in improving physical health among individuals undergoing compulsory rehabilitation, and identify key intervention parameters such as the optimal intervention cycle, frequency, and duration for related indicators, aiming to provide evidence-based support for the well-being in individuals undergoing compulsory rehabilitation.

## Methods

### Study design

This study was reported in accordance with the Cochrane Handbook for Systematic Reviews of Interventions and the Preferred Reporting Items for Systematic Reviews and Meta-Analyses (PRISMA) statement (11).The review was registered in PROSPERO (CRD420251029820).

### Information sources

Databases including Web of Science, PubMed, Cochrane Library, Medline, CNKI (China National Knowledge Infrastructure), Wanfang Data, and VIP Chinese Journal Database were searched (12).

### Search strategy

Literature retrieval was conducted using a combination of subject terms and free terms. When necessary, references of the included literature were also searched. For Chinese literature, subject terms such as “运动锻炼” (exercise training), “强戒人员” (individuals undergoing compulsory rehabilitation), “吸毒” (drug abuse), “戒毒” (drug rehabilitation), and “随机对照试验” (randomized controlled trials) were used for retrieval. For English literature, search terms such as “drug addicts” OR “Methamphetamine” OR “Drug User” AND “Exercise, Physical” AND “Physical Fitness” were used. Online searches were conducted in databases including Web of Science, PubMed, Cochrane Library, Medline, CNKI, Wanfang Data, and VIP Chinese Journal Database for randomized controlled trials related to exercise intervention on the physical health of individuals undergoing compulsory rehabilitation. The search covered the period from 2019 to December 2024, with languages restricted to English and Chinese.

The search was restricted to literature published between January 2019 and December 2024. This time frame was chosen to (1) capture the most current evidence reflecting contemporary exercise rehabilitation protocols (2), ensure consistency in the application of modern randomized controlled trial (RCT) reporting standards, and (3) provide clinically applicable recommendations for current drug rehabilitation practices. While this restriction may exclude older RCTs, it prioritizes the relevance and applicability of the findings to today’s compulsory rehabilitation settings.

### Inclusion and exclusion criteria

#### Eligibility criteria

1. The study type was published randomized controlled trials (RCTs).
2. Participants were compulsory drug rehabilitation individuals meeting international or domestic diagnostic criteria.
3. Studies provided quantifiable pre- and post-intervention data for both experimental and control groups.
4. Outcome measures included sit-and-reach, grip strength, single-leg standing with eyes closed, choice reaction time, or vital capacity.

#### Exclusion criteria for literature

1. Reviews, duplicate publications, and non-randomized controlled trials were excluded.
2. Studies were excluded if the research results were not quantified or lacked corresponding outcome measures.
3. Literature that could not be obtained in full text through various channels and methods was excluded.
4. Literature without a control group was excluded.

### Study selection

**Figure 1:**
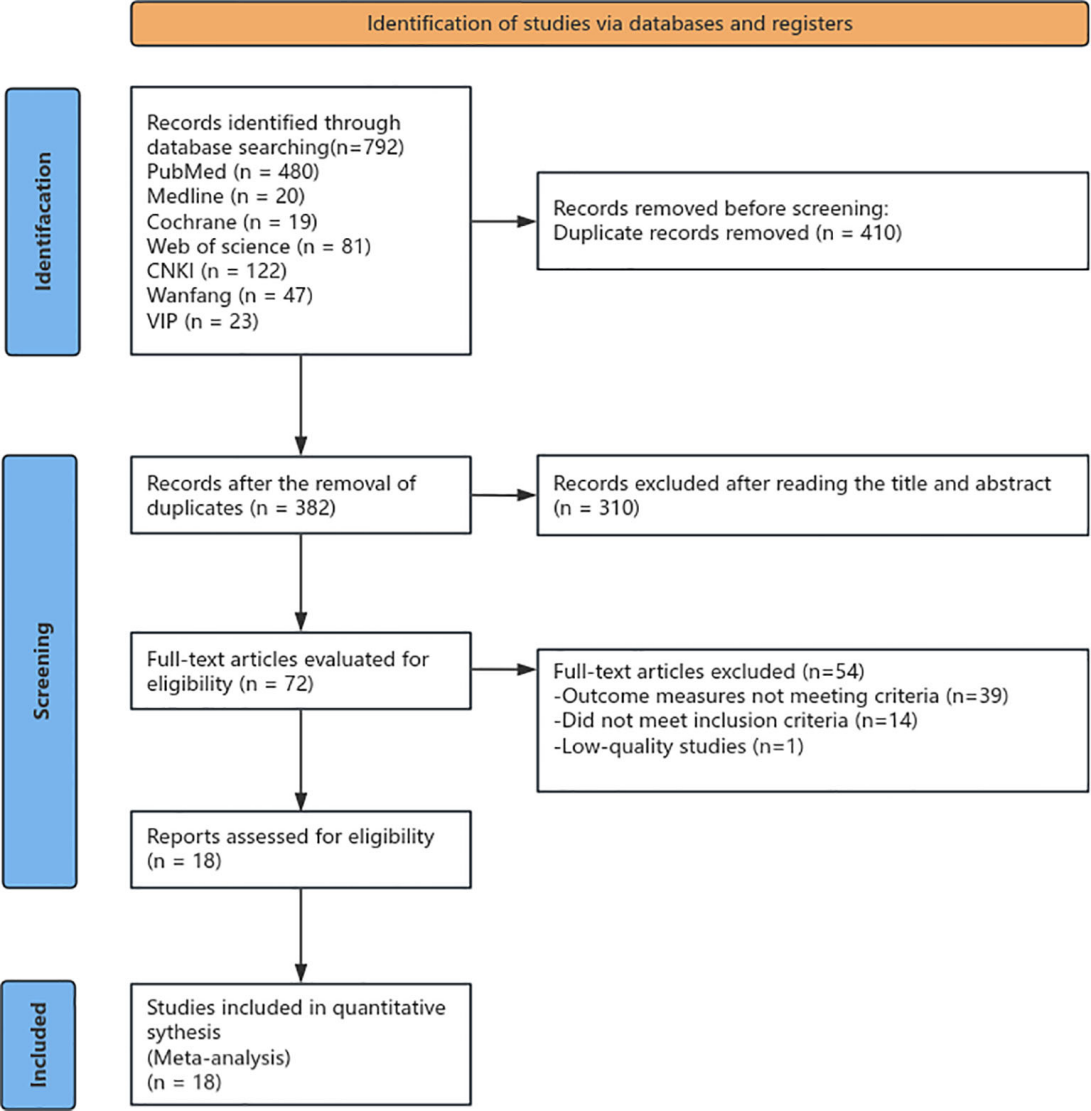
Preferred Reporting Items for Systematic Reviews and Meta-Analyses (PRISMA) flowchart.

**Figure 2:**
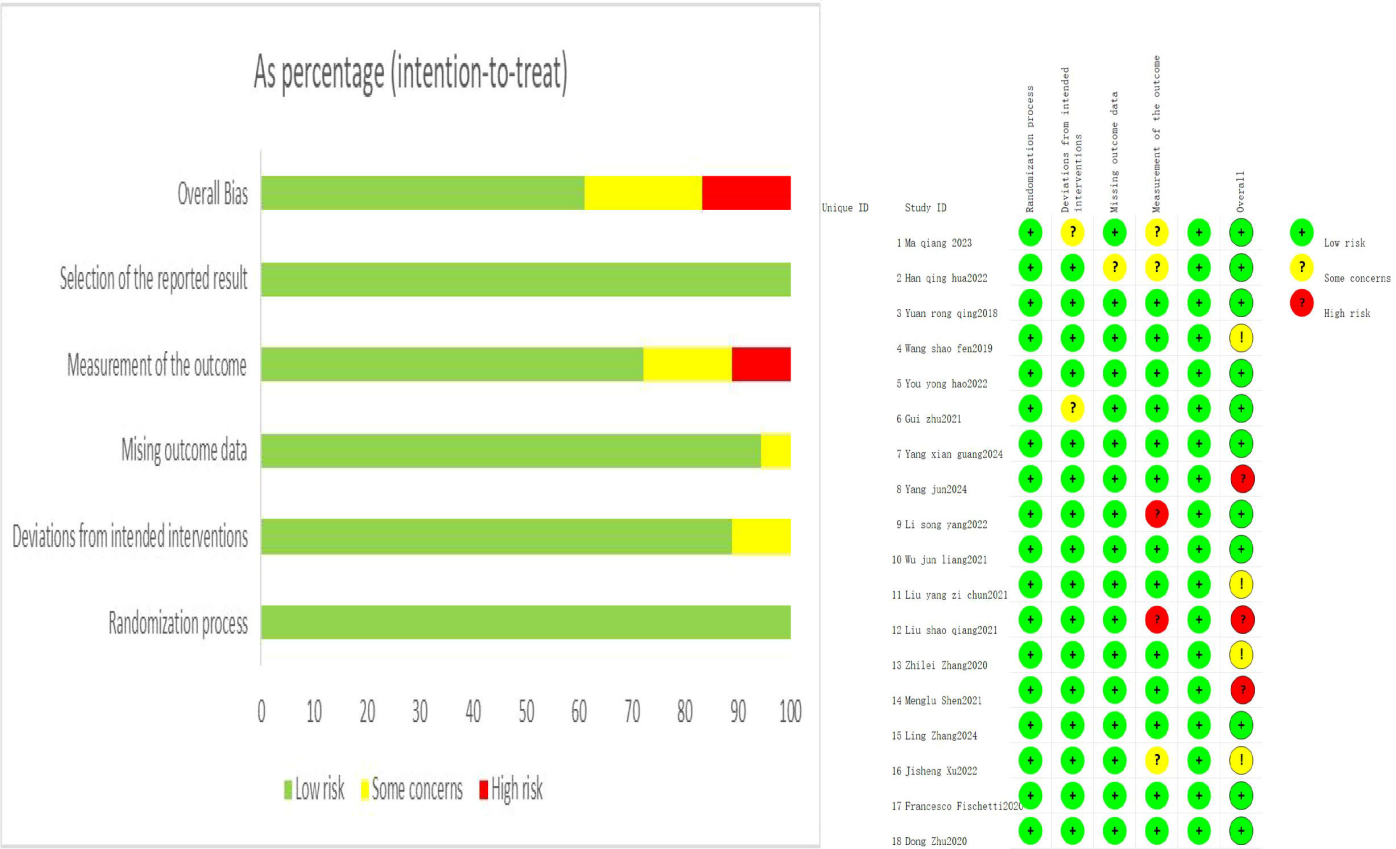
Literature bias risk assessment.

A total of 792 studies were initially retrieved, including 455 Chinese-language articles and 337 English-language articles. All retrieved literature was imported into NoteExpress software for management (13). First, two researchers independently conducted a quick review of the titles and abstracts of the retrieved literature. After initially screening by reading titles and abstracts and excluding duplicate literature, 410 studies relevant to this research were obtained. Subsequently, the full texts of these preliminarily selected studies were read in depth, and a comprehensive evaluation was conducted from multiple dimensions such as research methods, inclusion criteria, and outcome indicators. A further 54 studies that did not meet the established criteria were excluded. Finally, 18 articles were included in the Meta-analysis (see **Figures 1**, **2**).

### Data extraction

1. After reading the full texts, the two researchers extracted key information from the literature. The information included:
2. Basic information such as the name of the first author, the specific publication time, and the country of publication.
3. Specific details of intervention measures, such as different exercise modes, exercise cycles, exercise frequency, exercise duration, training intensity (e.g., %HRmax, RPE), and training structure (e.g., continuous vs. interval training, including sets, repetitions, and rest intervals where applicable).
4. Outcome indicators, focusing mainly on relevant data such as sit-and-reach, grip strength, single-leg stance with eyes closed, vital capacity, and choice reaction time.
5. In the process of data extraction, if any disputes arose, a third researcher was invited to participate in the discussion, and a consensus was reached through joint consultation.

### Risk of bias and evidence synthesis

The Cochrane Risk of Bias assessment tool built into RevMan 5.4 provided by the Cochrane Collaboration was adopted (14) to evaluate the methodological quality of the included literature. The evaluation results were categorized as “low risk,” “high risk,” or “unclear.” When there was a disagreement in the assessment, a third researcher intervened for consultation to decide whether to include the literature.

### Statistical analysis

Review Manager software (RevMan 5.4, Cochrane Collaboration, Oxford, UK) was used to perform effect size pooling, heterogeneity testing, subgroup analysis, and generate funnel plots. The mean difference was statistically analyzed using the mean difference (MD) and 95% confidence interval (CI). Funnel plots were used to assess publication bias, with a significance level of P = 0.05 (15). When I² ≤ 50% and P > 0.10, the heterogeneity among the included studies was low and the homogeneity was good, so a fixed-effects model was used for pooling. When 50% < I² ≤ 75%, indicating moderate heterogeneity, a random-effects model was applied for pooling. When I² > 75%, the heterogeneity was considered high. If the outcome measures of the literature showed a high level of heterogeneity, sensitivity analysis was conducted, and subgroup analysis was performed based on the sources of the heterogeneity.

Given the exploratory nature of this review and the number of planned subgroup analyses (exercise mode, frequency, duration, and cycle), the potential for inflated Type I error rates due to multiple comparisons was acknowledged. The primary aim of the meta-analysis was to determine the overall effect of exercise on each physical health outcome. Subgroup analyses were conducted to explore potential sources of heterogeneity and to generate hypotheses for optimal exercise prescription parameters. Therefore, the findings from subgroup comparisons should be interpreted with caution, and p-values from these analyses were considered descriptive rather than definitive confirmatory evidence.

## Results

### Literature search

A total of 18 articles were included in this study, all of which are randomized controlled trials. Among them, there were 822 participants in the experimental group and 816 in the control group, with a total sample size of 1,638. All participants were divided into the experimental group and the control group in accordance with the randomization principle. Intervention contents for the experimental group: 8 articles adopted aerobic exercise, 6 articles used traditional fitness exercise, 2 articles applied HIIT (High-Intensity Interval Training), 1 article employed physical functional exercise, and 1 article utilized gas volleyball exercise. Regarding exercise frequency: 9 articles reported a frequency of ≤ 3 days/week, and 9 articles had a frequency of > 3 days/week. For the exercise cycle: 11 articles had a cycle of ≤ 12 weeks, and 7 articles had a cycle of > 12 weeks. In terms of exercise duration: 6 articles specified a duration of ≤ 40 minutes, and 12 articles had a duration of > 40 minutes. Concerning the age of participants: 10 articles included participants aged ≤ 35 years, 8 articles involved those aged > 35 years, and 1 article did not specify the age in detail. The intervention measures for the control group included routine rehabilitation intervention, aerobics, recreational activities, and jogging (see **Table 1**).

**Table 1:** Study characteristics.

Author	Sample size (T/C)	Age (T/C)	Duration per session (min)	Frequency (sessions/week)	Intervention period (weeks)	Training intensity	Intervention parameters		Outcome indicators
							T	C	
Zhaoqiang Liu (16)	10/10	33.86 ± 2.9/ 35.12 ± 2.3	60/min	6	12	40-60%HRmax;RPE 11-13*(rehabilitation)*	12-week physical function training	12-week regular production work	Sit-and-reach (cm), single-leg standing with eyes closed (s), grip strength (kg)
Zichunliu Yang (17)	50/50	32.89 ± 6.23/ 34.53 ± 5.84	60/min	3	16	HIIT:80-90%HRmax (sprint)/40-50%HRmax (recovery);RPE 15-17/9-11	16-week High-Intensity Interval Training(HIIT)	Conventional rehabilitation therapy	Single-leg standing with eyes closed (s), choice reaction time (s), sit-and-reach (cm), grip strength (kg)
Rongqin Yuan (18)	30/30	30.5 ± 5.6/ 31.5 ± 6.0	30/min	3	12	Moderate intensity:50-60%HRmax;RPE 12-14	Moderate-intensity aerobic exercise	Tai Chi and aerobics training	Grip strength (kg), vital capacity (ml)
Gui Zhu (19)	34/33	64.23 ± 3.15/ 64.42 ± 2.91	120/min	5	24	Moderate intensity:45-55%HRmax;RPE 10-12*(elderly rehabilitation)*	Moderate-intensity aerobic exercise + health education guidance	Conventional drug rehabilitation health education and rehabilitation treatment	Vital capacity (ml), grip strength (kg), sit-and-reach (cm), single-leg standing with eyes closed (s), choice reaction time (s)
Junliang Wu (20)	30/30	33±4/ 33 ± 7	60/min	3	12	Aerobic:50-60%HRmax;Strength:60-70%1RMRPE 12-14/13-15	Moderate-intensity aerobic exercise + strength training	Conventional drug rehabilitation health education	Sit-and-reach (cm), choice reaction time (s), single-leg standing with eyes closed (s)
Jun Yang (21)	30/30	32.7 ± 4.27/ 34.6 ± 3.83	40/min	3	12	HIIT:75-85%HRmax (sprint)/40-50%HRmax (recovery);RPE 14-16/9-11	HIIT exercise	Conventional rehabilitation therapy	Single-leg standing with eyes closed (s)
Xiangguang Yang (22)	200/200	18 ± 59/ 18 ± 59	30/min	5	12	Moderate intensity:50-65%HRmax;RPE 12-14	Aerobic exercise + stationary cycling	Conventional rehabilitation therapy	Vital capacity (ml), grip strength (kg), sit-and-reach (cm), single-leg standing with eyes closed (s), choice reaction time (s)
Qiang Ma (23)	30/30	30.2 ± 5.4/ 30.9 ± 5.8	30/min	3	16	Traditional exercise:30-40%HRmax;Brisk walking:50-60%HRmaxRPE 8-10/12-14	Tai Chi exercise	Traditional physical fitness training (arranged aerobics)	Vital capacity (ml), grip strength (kg), sit-and-reach (cm), single-leg standing with one eye closed (s), choice reaction time (s)
Qinghua Han (24)	15/15	36.47 ± 6.32/ 36.47 ± 6.32	40/min	5	12	Moderate intensity:45-55%HRmax;RPE 11-13*(sports rehabilitation)*	Gas volleyball practice	Conventional drug rehabilitation health education	Grip strength (kg), single-leg standing with eyes closed (s), choice reaction time (s)
Shaofen Wang (25)	54/45	—	60/min	5	20	Traditional exercise:30-40%HRmax;RPE 8-10	Combination of Tai Chi	Conventional drug rehabilitation health education	Vital capacity (ml), sit-and-reach (cm), single-leg standing with eyes closed (s)
Songyang Li (26)	88/88	38.08 ± 6.54/ 37.25 ± 6.98	30/min	3	24	Aerobic:50-60%HRmax;Resistance:50-60%1RMRPE 12-14/12-14	Aerobic exercise + resistance training	Conventional drug rehabilitation health education	Vital capacity (ml), grip strength (kg), sit-and-reach (cm), single-leg standing with eyes closed (s)
Yonghao You (27)	61/59	29.4 ± 4.8/ 29.7 ± 5.4	60/min	5	20	Moderate intensity:50-65%HRmax;RPE 12-14	Moderate-intensity aerobic exercise	Conventional drug rehabilitation health education	Vital capacity (ml), grip strength (kg), sit-and-reach (cm), single-leg standing with eyes closed (s), choice reaction time (s)
Francesco Fischetti (28)	17/17	47.6 ± 12.1/ 42.9 ± 13.9	60/min	3	8	Aerobic:50-60%HRmax;Anaerobic:70-80%HRmaxRPE 12-14/14-16	Moderate-intensity aerobic	Conventional drug rehabilitation health education	Single-leg standing with eyes closed (s), sit-and-reach (cm)
Zhilei Zhang (29)	38/38	41.08 ± 9.94/ 39.11 ± 8.90	70/min	5	24	Traditional exercise:30-40%HRmax;RPE 8-10	Tai Chi exercise	Conventional drug rehabilitation health education	Vital capacity (ml), single-leg standing with eyes closed (s), grip strength (kg), sit-and-reach (cm)
Dong Zhu (30)	50/50	32 ± 5/ 30 ± 5	60/min	5	12	Traditional exercise:30-40%HRmax;RPE 8-10	Tai Chi exercise	Control group: recreational activities	Sit-and-reach (cm), single-leg standing with eyes closed (s), vital capacity (ml)
Menglu Shen (31)	35/37	39.31 ± 10.33/ 39.37 ± 9.28	60/min	3	12	Traditional exercise:30-40%HRmax;RPE 8-10	Tai Chi exercise	Conventional drug rehabilitation health education	Vital capacity (ml), grip strength (kg), sit-and-reach (cm), single-leg standing with one eye closed (s)
Jisheng Xu (32)	30/30	31.30 ± 3.86/ 29.50 ± 4.59	60/min	5	12	Moderate intensity:50-65%HRmax;RPE 12-14	Moderate-intensity aerobic exercise	Conventional drug rehabilitation health education	Vital capacity (ml), grip strength (kg), sit-and-reach (cm), choice reaction time (s)
Ling Zhang (33)	20/24	38.30 ± 9.07/ 39.70 ± 8.56	60/min	3	12	Tai Chi:30-40%HRmax (RPE8-10);Aerobic (control):50-60%HRmax (RPE12-14)	Tai Chi practice	Moderate-intensity aerobic exercise	Vital capacity (ml), grip strength (kg), single-leg standing with eyes closed (s), choice reaction time (s)

T, experimental group; C, control group; HIIT, High-Intensity Interval Training; %HRmax, percentage of maximum heart rate; %1RM, percentage of one-repetition maximum; RPE, Rating of Perceived Exertion (6–20 scale); —, data not reported in the original study.

### Quality assessment of included studies

The 18 included studies were assessed using the Cochrane Risk of Bias (ROB 2.0) tool. The domains of randomization process and selection of reported results were uniformly rated as low risk. Deviations from intended interventions and missing outcome data were also predominantly low risk, with only a small subset of studies raising some concerns. Outcome measurement represented the primary source of bias, with several studies exhibiting some concerns or high risk. Overall, most studies were at low risk of bias or had minor concerns, supporting the reliability of the synthesized findings, although high-risk studies warrant cautious interpretation (**Figure 2**)

### Meta-analysis results

#### Effect of exercise intervention on sit-and-reach

**Figure 3:**
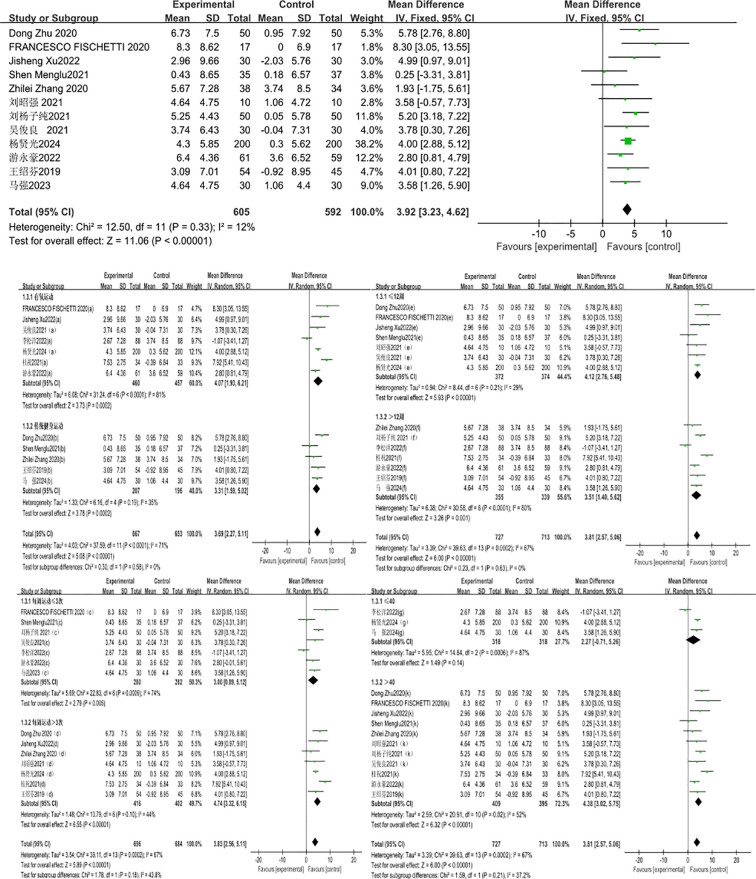
Forest plot of the effect of exercise intervention on sit-and-reach of individuals undergoing compulsory drug rehabilitation.

A total of 12 studies were included with the sit-and-reach test as the indicator. The 12 studies showed low heterogeneity (I²=12%, P = 0.33), so a fixed-effects model was used for pooling. The results showed that the effect of exercise intervention on the sit-and-reach performance of compulsory detoxification personnel was significantly better than that of the control group, with a statistically significant difference (MD = 3.92, 95%CI = 3.23, 4.62, P < 0.00001) (**Figure 3**).

The results of subgroup analysis showed that a total of 12 studies involving 1,320 subjects were included. Under the random-effects model, the aerobic exercise mode had a significantly better improvement effect on the sit-and-reach performance of compulsory detoxification personnel than the traditional fitness exercise mode. There was high heterogeneity in the difference in effect sizes between the two groups (I²=71%), indicating that the exercise mode has a significant moderating effect on the intervention outcome. Among them, the heterogeneity test of the aerobic exercise group showed that: X²=31.24, I²=81%, P<0.001, with a pooled effect size of MD = 4.07, 95%CI=1.93~6.21, P = 0.0002, indicating a statistically significant difference. The traditional fitness exercise group showed that: X²=6.16, I²=35%, P = 0.19, with a pooled effect size of MD = 3.13, 95%CI=1.59~5.02, P = 0.0002, indicating a statistically significant difference.

The exercise cycles were divided into two categories: ≤ 12 weeks and > 12 weeks, and subgroup analysis was conducted for these two cycles. A total of 14 studies involving 1,440 subjects were included. There was high heterogeneity in the difference in effect sizes between the two groups (I²=80%), indicating that the exercise cycle has a significant moderating effect on the intervention outcome. Among them, the heterogeneity test for the ≤ 12-week subgroup showed: X²=8.44, I²=29%, P = 0.21, with a pooled effect size of MD = 4.12, 95%CI=2.76~5.48, P<0.0001, indicating a statistically significant difference. For the > 12-week subgroup: X²=30.58, I²=80%, P<0.0001, with a pooled effect size of MD = 3.51, 95%CI=1.40~5.62, P = 0.001, also showing a statistically significant difference. It can be seen that the ≤ 12-week subgroup has the largest effect size.

The exercise frequency was divided into two subgroups: ≤ 3 sessions per week and > 3 sessions per week, and subgroup analysis was conducted accordingly. A total of 14 studies involving 1,380 subjects were included, and subgroup analysis was performed based on these two frequency categories. There was high heterogeneity in the difference in effect sizes between the two groups (I²=67%), indicating that exercise frequency has a significant moderating effect on the intervention outcome. Among them, the heterogeneity test for the subgroup with ≤ 3 sessions per week showed: X²=22.83, I²=74%, P = 0.0009, with a pooled effect size of MD = 3.00, 95%CI=0.89~5.12, P = 0.005, indicating a statistically significant difference. For the subgroup with > 3 sessions per week: X²=10.79, I²=44%, P = 0.10, with a pooled effect size of MD = 4.74, 95%CI=3.32~6.15, P<0.00001, also showing a statistically significant difference. Moreover, the effect size was the most significant in the subgroup with > 3 sessions per week.

The exercise duration was divided into two subgroups: ≤ 40 minutes and > 40 minutes, and subgroup analysis was conducted accordingly. A total of 14 studies involving 1,440 subjects were included. There was high heterogeneity in the difference in effect sizes between the two groups (I²=67%), indicating that exercise duration has a significant moderating effect on the intervention outcome. Among them, the heterogeneity test for the ≤ 40-minute subgroup showed: X²=14.84, I²=87%, P = 0.0006, with a pooled effect size of MD = 2.27, 95%CI=-0.71~5.26, P = 0.14, indicating no statistically significant difference. For the > 40-minute subgroup, the heterogeneity test showed: X²=20.91, I²=52%, P = 0.02, with a pooled effect size of MD = 4.38, 95%CI=3.02~5.75, P<0.00001, indicating a statistically significant difference.

### Effect of exercise intervention on single-leg standing with eyes closed

**Figure 4:**
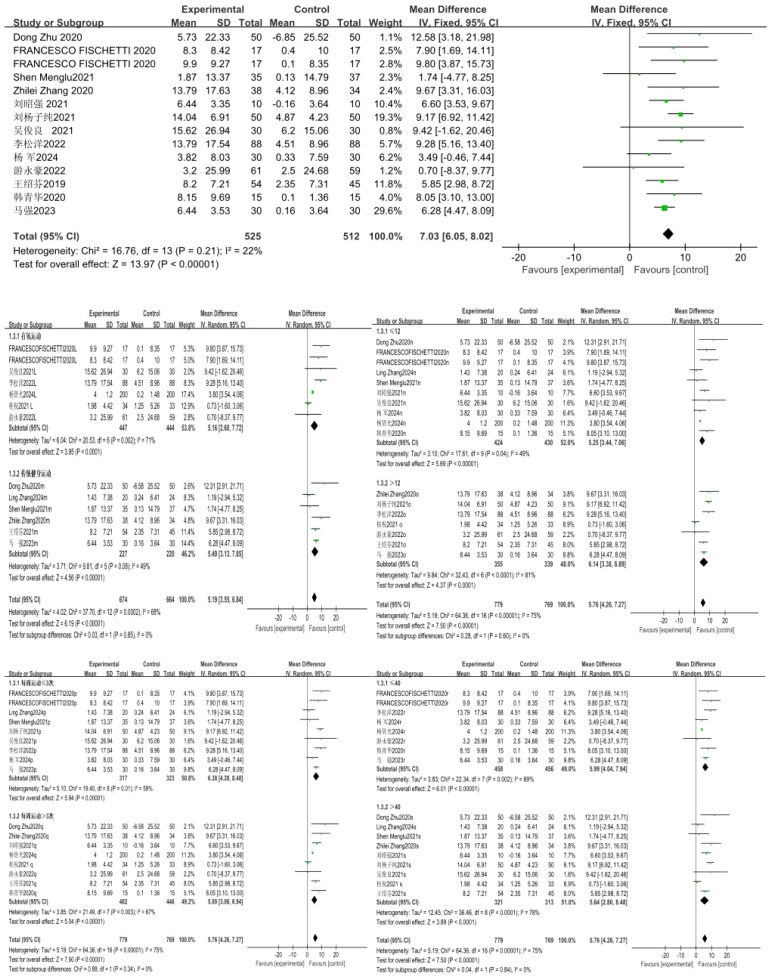
Forest plot of the effect of exercise intervention on single-leg standing with eyes closed of individuals undergoing compulsory drug rehabilitation.

A total of 13 studies were included, with the one-leg standing with eyes closed test as the indicator. The 13 studies showed low heterogeneity (I²=22%, P = 0.21), so a fixed-effects model was used for pooling. The results showed that the effect of exercise intervention on the one-leg standing with eyes closed performance of compulsory detoxification personnel was significantly better than that of the control group, with a statistically significant difference (MD = 7.03, 95%CI=6.05, 8.02, P<0.00001) (**Figure 4**).

The results of the subgroup analysis showed that a total of 12 studies involving 1,338 subjects were included in this group. Under the random-effects model, traditional fitness exercise had a significantly better improvement effect on the one-leg standing with eyes closed performance of compulsory detoxification personnel than aerobic exercise. There was high heterogeneity in the difference in effect sizes between the two groups (I²=68%), indicating that the exercise mode has a significant moderating effect on the intervention outcome. Among them, the heterogeneity test of the aerobic exercise group showed that: X²=20.53, I²=71%, P = 0.002, with a pooled effect size of MD = 5.16, 95%CI=2.60~7.72, P<0.0001, indicating a statistically significant difference; the traditional fitness exercise group showed that: X²=9.81, I²=49%, P = 0.08, with a pooled effect size of MD = 5.49, 95%CI=3.31~7.85, P<0.00001, indicating a statistically significant difference, and the effect size of traditional fitness exercise was more significant.

The exercise cycles were divided into two categories: ≤ 12 weeks and > 12 weeks, and subgroup analysis was conducted for these two cycles. A total of 16 studies involving 1,548 subjects were included in this group. There was high heterogeneity in the difference in effect sizes between the two groups (I²=75%), indicating that the exercise cycle has a significant moderating effect on the intervention outcome. Under the random-effects model, the heterogeneity test for the ≤ 12-week subgroup showed: X²=17.61, I²=49%, P = 0.04, with a pooled effect size of MD = 5.25, 95%CI=3.44~7.06, P<0.00001, indicating a statistically significant difference; the > 12-week subgroup showed: X²=32.43, I²=81%, P<0.0001, with a pooled effect size of MD = 6.14, 95%CI=3.38~8.89, P<0.0001, also indicating a statistically significant difference. Moreover, the effect size of exercise lasting > 12 weeks was superior to that of exercise lasting ≤ 12 weeks.

The exercise frequencies were divided into two categories: ≤ 3 sessions per week and > 3 sessions per week, and subgroup analysis was conducted for these two frequencies. A total of 16 studies involving 1,548 subjects were included in this group. There was high heterogeneity in the difference in effect sizes between the two groups (I²=75%), indicating that exercise frequency has a significant moderating effect on the intervention outcome. Under the random-effects model, the heterogeneity test for the subgroup with ≤ 3 exercise sessions per week showed: X²=19.04, I²=59%, P = 0.01, with a pooled effect size of MD = 6.38, 95%CI=4.28~8.48, P<0.00001, indicating a statistically significant difference; the subgroup with > 3 exercise sessions per week showed: X²=21.49, I²=67%, P = 0.003, with a pooled effect size of MD = 5.00, 95%CI=3.06~6.94, P<0.00001, also indicating a statistically significant difference. It can be seen that the effect size of exercising ≤ 3 times per week is more significant.

The exercise duration was divided into two categories: ≤ 40 minutes and > 40 minutes, and subgroup analysis was conducted for these two durations. A total of 16 studies involving 1,548 subjects were included in this group. There was high heterogeneity in the difference in effect sizes between the two groups (I²=75%), indicating that exercise duration has a significant moderating effect on the intervention outcome. Under the random-effects model, the heterogeneity test for the ≤ 40-minute subgroup showed: X²=22.34, I²=69%, P = 0.002, with a pooled effect size of MD = 5.99, 95%CI=4.04~7.94, P<0.00001, indicating a statistically significant difference; the heterogeneity test for the > 40-minute subgroup showed: X²=36.46, I²=78%, P<0.0001, with a pooled effect size of MD = 5.64, 95%CI=2.80~8.48, P<0.0001, also indicating a statistically significant difference. Moreover, the effect size of exercise duration ≤ 40 minutes was more significant.

### Effect of exercise intervention on grip strength

**Figure 5:**
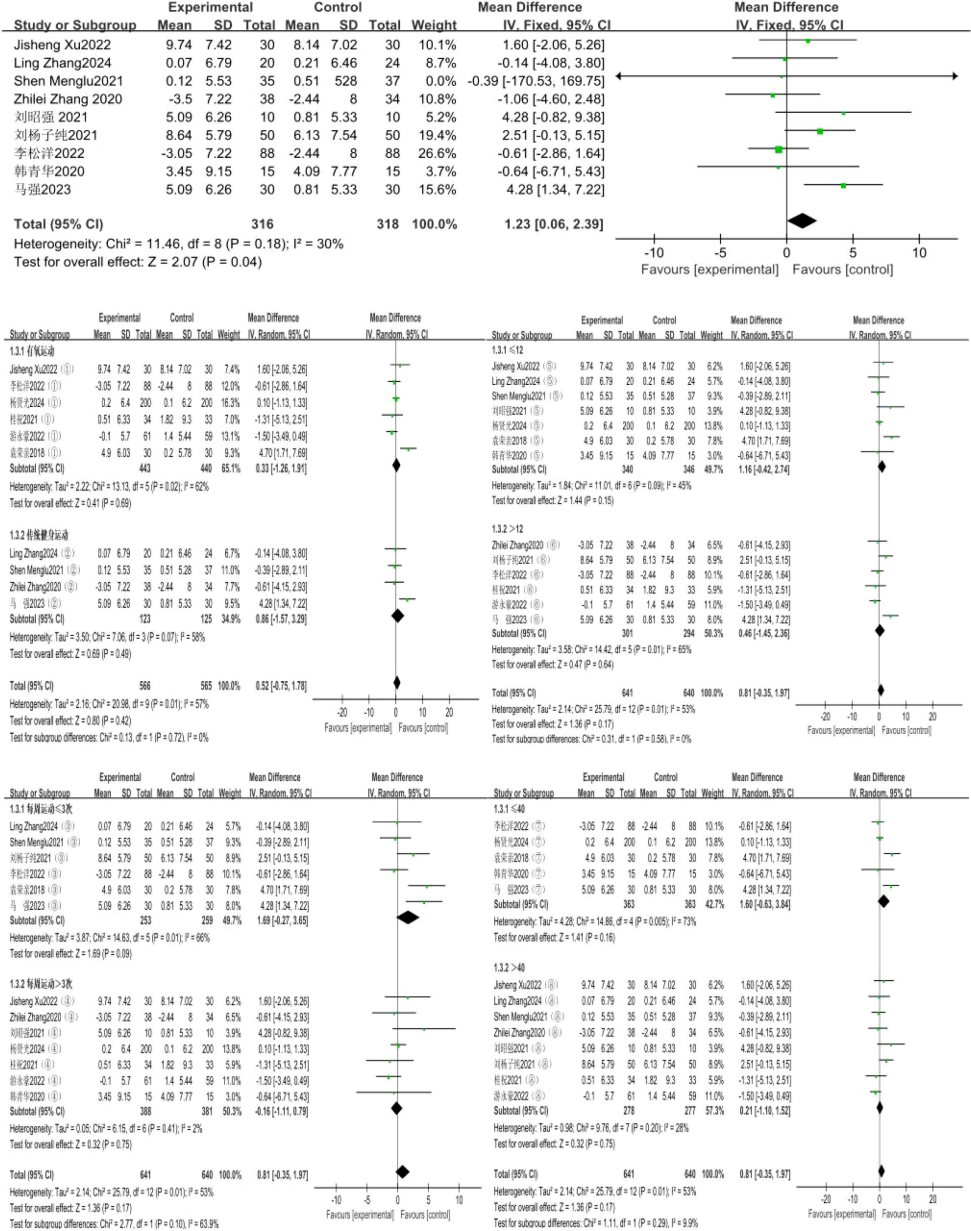
Forest plot of the effect of exercise intervention on grip strength of individuals undergoing compulsory drug rehabilitation.

A total of 9 studies were included, with grip strength as the indicator. The 9 studies showed low heterogeneity (I²=30%, P = 0.18), so a fixed-effects model was used for analysis. The results showed that the effect of exercise intervention on the grip strength of compulsory detoxification personnel was significantly better than that of the control group, with a statistically significant difference (MD = 1.23, 95%CI=0.06, 2.39, P = 0.04) (**Figure 5**).

The results of the subgroup analysis showed that a total of 10 studies involving 2,014 subjects were included in this group. There was high heterogeneity in the difference in effect sizes between the two groups (I²=57%), indicating that the exercise mode has a significant moderating effect on the intervention outcome. Under the random-effects model, the heterogeneity test of the aerobic exercise group showed: X²=13.13, I²=62%, P = 0.02, with a pooled effect size of MD = 0.33, 95%CI=-1.26~1.91, P = 0.69, indicating no statistically significant difference; the traditional fitness exercise group showed: X²=7.06, I²=58%, P = 0.08 (Note: There might be a typo in the original text where “P=7.06” is incorrect, and it is revised here based on statistical logic), with a pooled effect size of MD = 0.86, 95%CI=-0.75~3.29, P = 0.19, also indicating no statistically significant difference.

The exercise cycles were divided into two categories: ≤ 12 weeks and > 12 weeks, and subgroup analysis was conducted for these two cycles. A total of 13 studies involving 1,281 subjects were included in this group. There was high heterogeneity in the difference in effect sizes between the two groups (I²=65%), indicating that the exercise cycle has a significant moderating effect on the intervention outcome. Under the random-effects model, the heterogeneity test for the ≤ 12-week subgroup showed: X²=11.01, I²=45%, P = 0.09, with a pooled effect size of MD = 1.16, 95%CI=-0.42~2.74, P = 0.15, indicating no statistically significant difference; the > 12-week subgroup showed: X²=14.42, I²=65%, P = 0.01, with a pooled effect size of MD = 0.46, 95%CI=-1.45~2.36, P = 0.64, also indicating no statistically significant difference.

The exercise frequencies were divided into two categories: ≤ 3 sessions per week and > 3 sessions per week, and subgroup analysis was conducted for these two frequencies. A total of 13 studies involving 121 subjects were included in this group. There was high heterogeneity in the difference in effect sizes between the two groups (I²=53%), indicating that exercise frequency has a significant moderating effect on the intervention outcome. Under the random-effects model, the heterogeneity test for the subgroup with ≤ 3 exercise sessions per week showed: X²=14.63, I²=66%, P = 0.01, with a pooled effect size of MD = 1.69, 95%CI=-0.27~3.65, P = 0.09, indicating no statistically significant difference; the subgroup with > 3 exercise sessions per week showed: X²=6.15, I²=2%, P = 0.41, with a pooled effect size of MD=-0.16, 95%CI=-1.11~0.79, P = 0.75, also indicating no statistically significant difference.

The exercise duration was divided into two categories: ≤ 40 minutes and > 40 minutes, and subgroup analysis was performed for these two time durations. A total of 13 studies involving 1,281 subjects were included in this group. There was high heterogeneity in the difference in effect sizes between the two groups (I²=53%), indicating that exercise duration has a significant moderating effect on the intervention outcome. Under the random-effects model, the heterogeneity test for the ≤ 40-minute subgroup showed: X²=14.86, I²=73%, P = 0.005, with a pooled effect size of MD = 1.60, 95%CI=-0.63~3.84, P = 0.16, indicating no statistically significant difference; the heterogeneity test for the > 40-minute subgroup showed: X²=9.76, I²=28%, P = 0.20, with a pooled effect size of MD = 0.21, 95%CI=-1.10~1.52, P = 0.75, also indicating no statistically significant difference.

### Effect of exercise intervention on vital capacity of compulsory detoxification personnel

**Figure 6:**
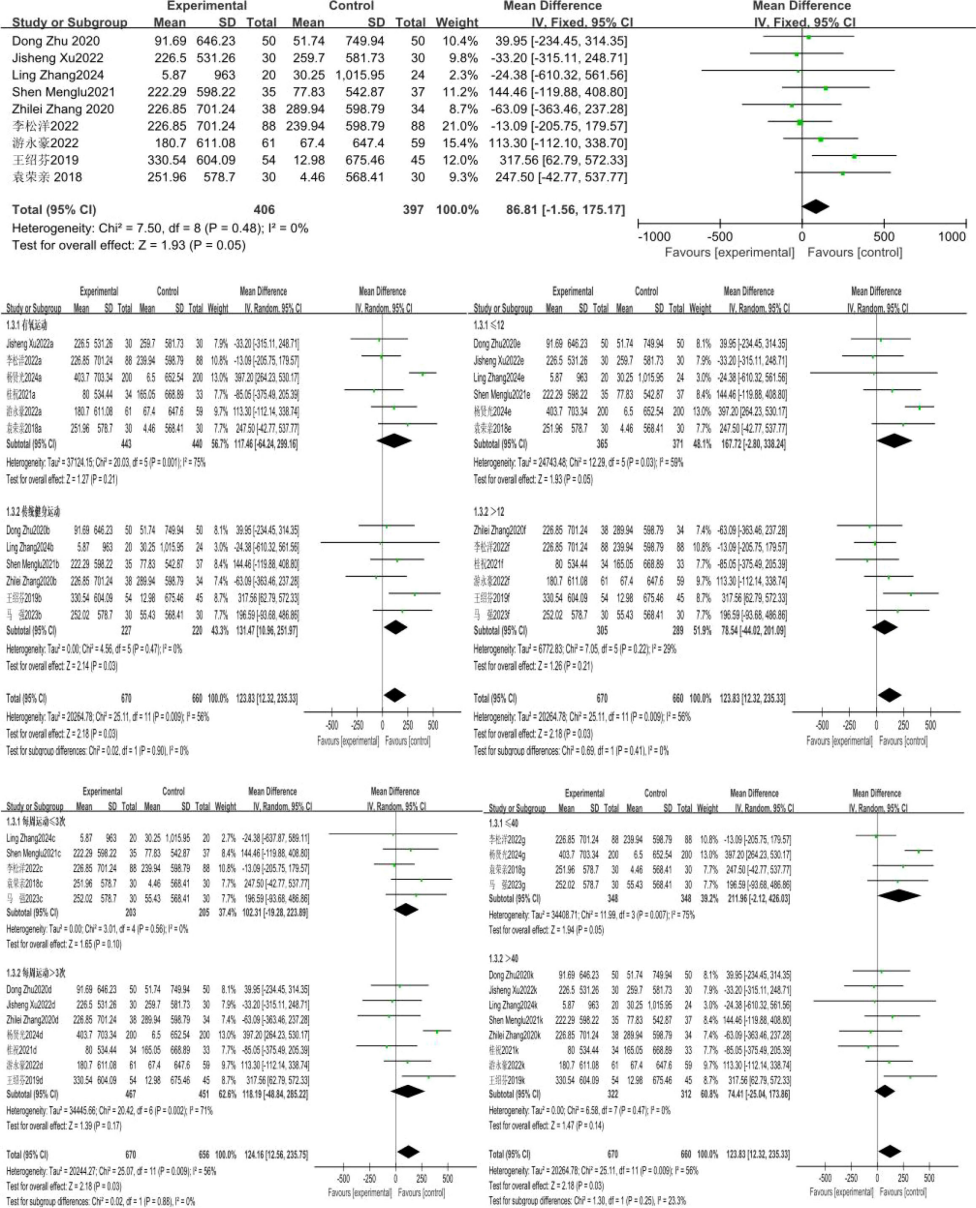
Forest plot of the effect of exercise intervention on vital capacity of individuals undergoing compulsory drug rehabilitation.

A total of 9 studies were included, with vital capacity as the indicator. The 9 studies exhibited low heterogeneity (I²=0%, P = 0.48), so a fixed-effects model was applied for analysis. The results showed that while the exercise intervention led to a positive trend in vital capacity for compulsory detoxification personnel compared to the control group, this difference did not reach statistical significance (MD = 86.81, 95%CI=-1.56, 175.17, P = 0.05) (**Figure 6**).

The results of the subgroup analysis showed that a total of 12 studies involving 1,330 subjects were included in this group. There was high heterogeneity in the difference in effect sizes between the two groups (I²=56%), indicating that the exercise mode has a significant moderating effect on the intervention outcome. Under the random-effects model, the heterogeneity test of the aerobic exercise group showed: X²=20.03, I²=75%, P = 0.001, with a pooled effect size of MD = 117.46, 95%CI=-64.24~299.16, P = 0.21, indicating no statistically significant difference; the traditional fitness exercise group showed: X²=4.56, I²=0%, P = 0.47, with a pooled effect size of MD = 131.47, 95%CI=10.96~251.97, P = 0.03, indicating a statistically significant difference.

The exercise cycles were divided into two subgroups: ≤ 12 weeks and > 12 weeks, and subgroup analysis was conducted for these two cycle categories. A total of 12 studies involving 1,330 subjects were included in this group. There was high heterogeneity in the difference in effect sizes between the two groups (I²=56%), indicating that the exercise cycle has a significant moderating effect on the intervention outcome. Under the random-effects model, the heterogeneity test for the ≤ 12-week subgroup showed: X²=12.29, I²=59%, P = 0.03, with a pooled effect size of MD = 167.72, 95%CI=-2.80~338.24, P = 0.05, indicating a statistically significant difference; the > 12-week subgroup showed: X²=7.05, I²=29%, P = 0.22, with a pooled effect size of MD = 78.54, 95%CI=-44.02~201.09, P = 0.21, indicating no statistically significant difference.

The exercise frequencies were divided into two subgroups: ≤ 3 sessions per week and > 3 sessions per week, and subgroup analysis was conducted for these two frequency categories. A total of 12 studies involving 1,326 subjects were included in this group. There was high heterogeneity in the difference in effect sizes between the two groups (I²=56%), indicating that exercise frequency has a significant moderating effect on the intervention outcome. Under the random-effects model, the heterogeneity test for the subgroup with ≤ 3 exercise sessions per week showed: X²=3.01, I²=0%, P = 0.56, with a pooled effect size of MD = 102.31, 95%CI=-19.28~223.89, P = 0.10, indicating no statistically significant difference; the subgroup with > 3 exercise sessions per week showed: X²=20.42, I²=71%, P = 0.002, with a pooled effect size of MD = 118.19, 95%CI=-48.84~285.22, P = 0.17, also indicating no statistically significant difference.

The exercise duration was divided into two subgroups: ≤ 40 minutes and > 40 minutes, and subgroup analysis was conducted for these two time categories. A total of 12 studies involving 1,330 subjects were included in this group. There was high heterogeneity in the difference in effect sizes between the two groups (I²=56%), indicating that exercise duration has a significant moderating effect on the intervention outcome. Under the random-effects model, the heterogeneity test for the ≤ 40-minute subgroup showed: X²=11.99, I²=79%, P = 0.007, with a pooled effect size of MD = 211.96, 95%CI=-2.12~426.03, P = 0.05, indicating a statistically significant difference; the heterogeneity test for the > 40-minute subgroup showed: X²=6.58, I²=0%, P = 0.47, with a pooled effect size of MD = 74.41, 95%CI=-25.04~173.86, P = 0.14, indicating no statistically significant difference (**Figures 8**, **9**).

### Effect of exercise intervention on choice reaction time

**Figure 7:**
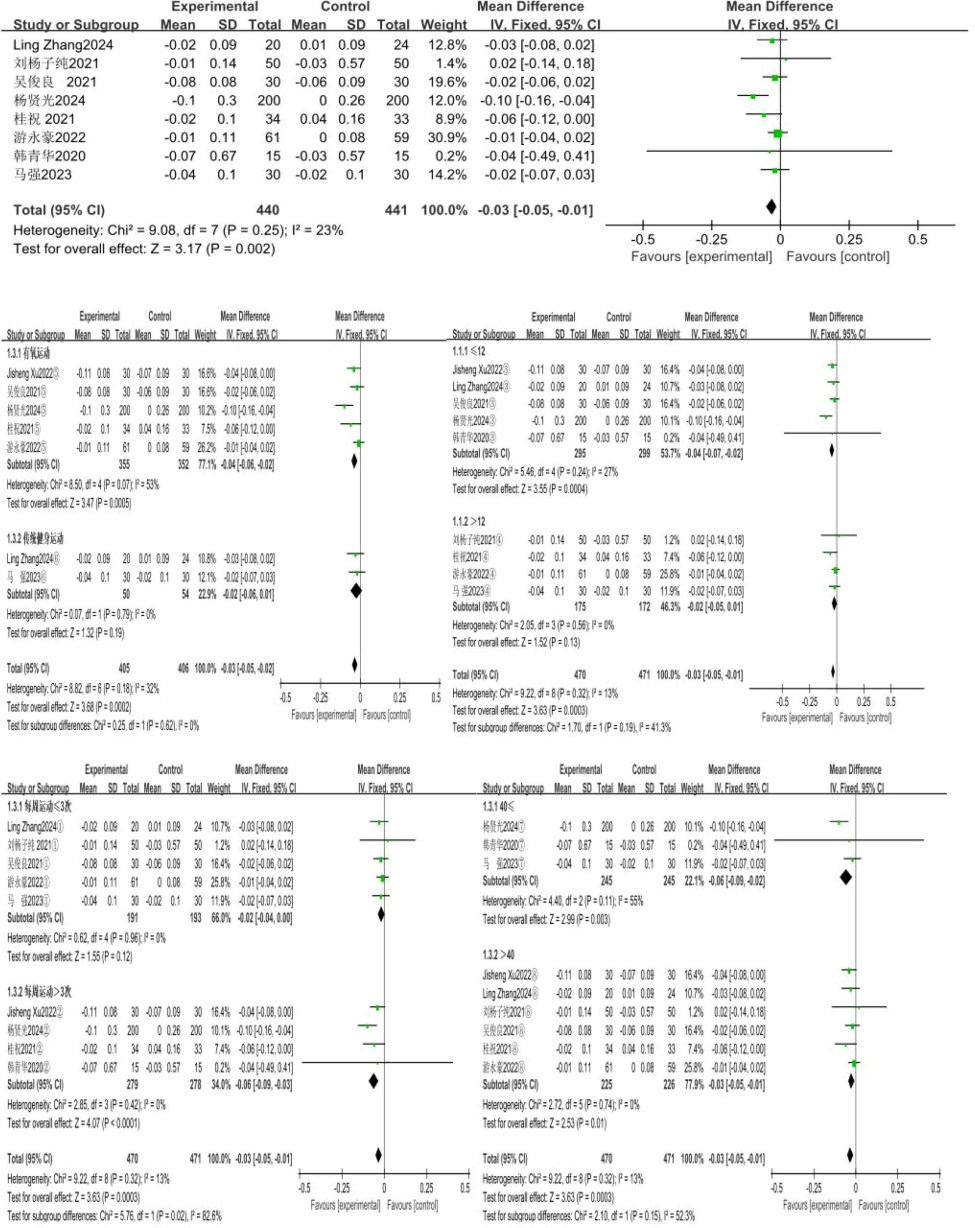
Forest plot of the effect of exercise intervention on choice reaction time of individuals undergoing compulsory drug rehabilitation.

A total of 8 studies were included, with choice reaction time as the indicator. The 8 studies showed low heterogeneity (I²=23%, P = 0.25), so a fixed-effects model was used for analysis. The results showed that the effect of exercise intervention on the choice reaction time of compulsory detoxification personnel was significantly better than that of the control group, with a statistically significant difference (MD=-0.03, 95%CI=-0.05, -0.01, P = 0.002) (**Figure 7**).

**Figure 8:**
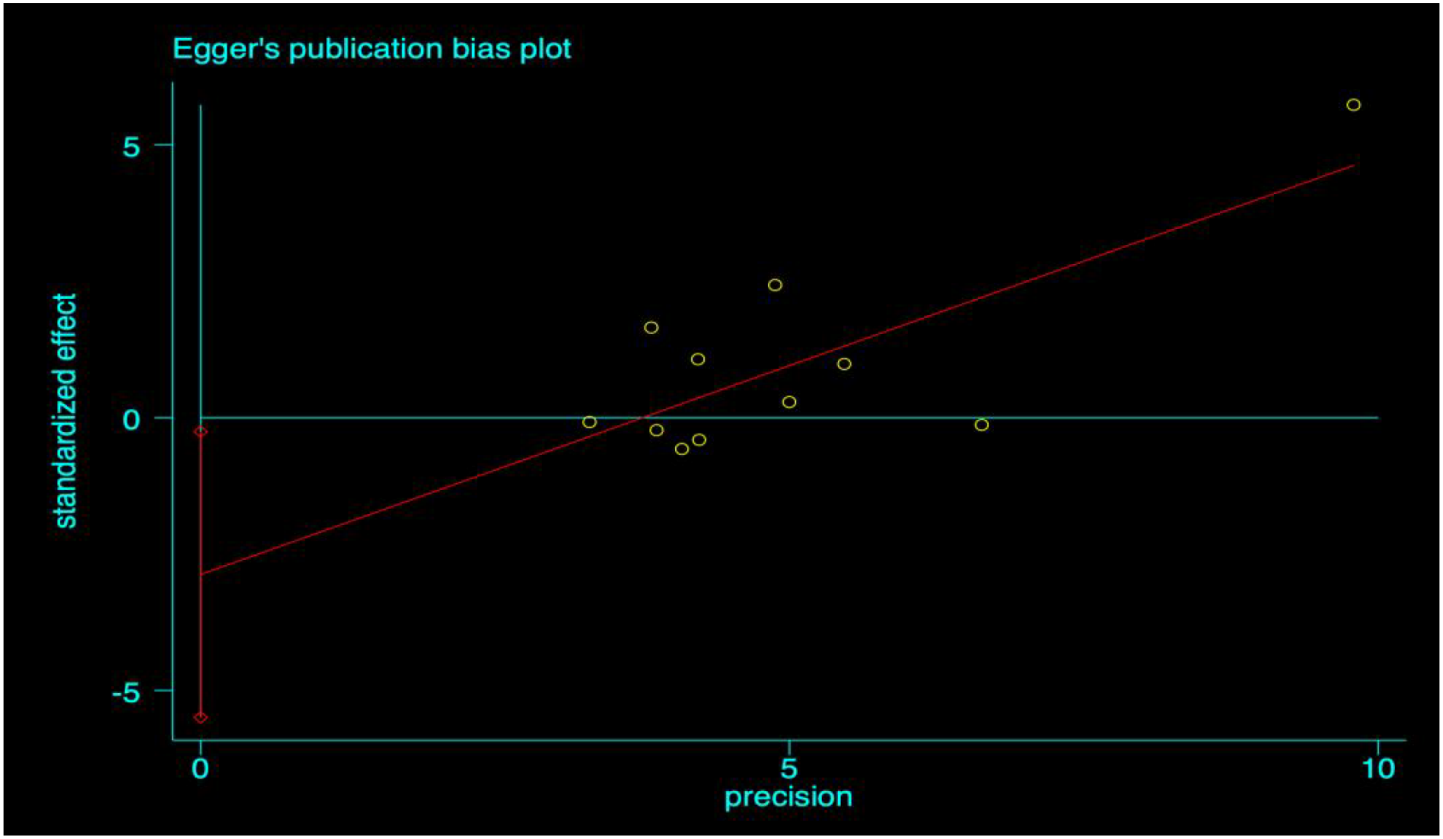
Egger’s test plot for vital capacity.

**Figure 9:**
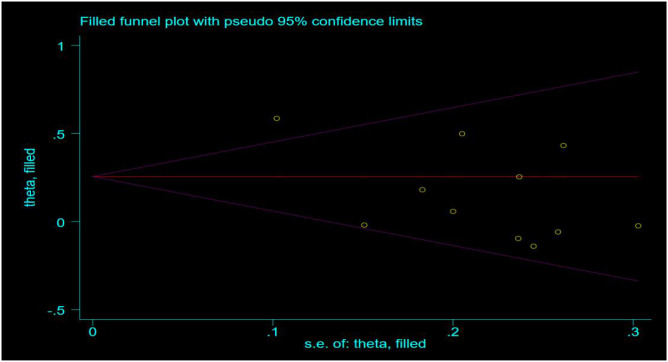
Vital capacity and trim-and-fill method.

The results of the subgroup analysis showed that a total of 7 studies involving 811 subjects were included in this group. There was moderate heterogeneity in the difference in effect sizes between the two groups (I²=32%).Under the fixed-effects model, the heterogeneity test of the aerobic exercise group showed: X²=8.50, I²=53%, P = 0.007, with a pooled effect size of MD=-0.04, 95%CI=-0.06~-0.02, P = 0.0005, indicating a statistically significant difference; the traditional fitness exercise group showed: X²=0.07, I²=0%, P = 0.79, with a pooled effect size of MD=-0.02, 95%CI=-0.06~-0.01, P = 0.19, indicating no statistically significant difference.

The exercise cycles were divided into two subgroups: ≤12 weeks and >12 weeks, and subgroup analysis was conducted accordingly. A total of 9 studies involving 941 subjects were included in this group. There was low heterogeneity in the difference in effect sizes between the two groups (I²=13%).Under the fixed-effects model, the heterogeneity test for the ≤ 12-week subgroup showed: X²=5.46, I²=27%, P = 0.24, with a pooled effect size of MD=-0.04, 95%CI=-0.07~-0.02, P = 0.0004, indicating a statistically significant difference; the > 12-week subgroup showed: X²=2.05, I²=0%, P = 0.56, with a pooled effect size of MD=-0.03, 95%CI=-0.05~-0.01, P = 0.13, indicating no statistically significant difference.

The exercise frequencies were divided into two subgroups: ≤ 3 sessions per week and > 3 sessions per week, and subgroup analysis was performed for these two frequency categories. A total of 9 studies involving 941 subjects were included in this group. There was good homogeneity in the difference in effect sizes between the two groups (I²=13%).Under the fixed-effects model, the heterogeneity test for the subgroup with ≤ 3 exercise sessions per week showed: X²=0.62, I²=0%, P = 0.96, with a pooled effect size of MD=-0.02, 95%CI=-0.04~0.00, P = 0.12, indicating no statistically significant difference; the subgroup with > 3 exercise sessions per week showed: X²=2.85, I²=0%, P = 0.42, with a pooled effect size of MD=-0.06, 95%CI=-0.09~-0.03, P<0.0001, indicating a statistically significant difference.

The exercise duration was divided into two subgroups: ≤ 40 minutes and > 40 minutes, and subgroup analysis was conducted for these two time categories. A total of 9 studies involving 941 subjects were included in this group. There was good homogeneity in the difference in effect sizes between the two groups (I²=13%).Under the fixed-effects model, the heterogeneity test for the ≤ 40-minute subgroup showed: X²=4.40, I²=55%, P = 0.11, with a pooled effect size of MD=-0.06, 95%CI=-0.09~-0.02, P = 0.003, indicating a statistically significant difference; the heterogeneity test for the > 40-minute subgroup showed: X²=2.72, I²=0%, P = 0.74, with a pooled effect size of MD=-0.03, 95%CI=-0.05~-0.01, P = 0.0003, indicating a statistically significant difference, and the effect size of exercise duration > 40 minutes was more significant.

### Sensitivity analysis

To assess the robustness of the meta-analysis results, this study conducted a sensitivity analysis of all included studies using the leave-one-out method. By omitting one study at a time and recalculating the pooled effect size, we observed whether the results fluctuated significantly. The analysis showed that when any single study was excluded, the pooled effect sizes of all outcome indicators did not change substantially, and the direction and statistical significance of the effect sizes remained consistent with those of the overall analysis. This indicates that the main conclusions of this study are robust and reliable, and are less affected by the results of any individual study.

### Publication bias analysis

This study employed the Egger test and trim-and-fill method to evaluate publication bias for five physical fitness test indicators: single-leg standing with eyes closed, vital capacity, grip strength, choice reaction time, and sit-and-reach. The results showed no statistically significant differences in the Egger test for single-leg standing with eyes closed, grip strength, choice reaction time, and sit-and-reach (P>0.05), indicating no significant publication bias in the combined analysis of these indicators. Only the Egger test for vital capacity revealed a statistically significant difference (P<0.05), suggesting a certain degree of publication bias. However, after correction with the trim-and-fill method, no significant bias was observed. Overall, the results of this study were minimally affected by publication bias, and the conclusions are highly reliable.

### Grading of recommendations assessment, development and evaluation

The GRADE (Grading of Recommendations Assessment, Development and Evaluation) system was employed to assess the quality of evidence for this meta-analysis, with evaluation focusing on five key dimensions: risk of bias, inconsistency, indirectness, imprecision, and publication bias. The quality of evidence was classified into four levels: high, medium, low, and very low. Among these, the evidence quality for sit-and-reach, closed-eye single-leg standing, and choice reaction time was rated as medium, while that for grip strength and vital capacity was rated as low. See **Table 2**.

**Table 2:** GRADE evidence quality rating scale for the impact of exercise interventions on the health of compulsory isolation personnel.

Outcome indicator	Effect size (MD, 95%CI)	P-value	Quality of evidence grade	Key downgrading reasons
Sit-and-reach	3.92 (3.23, 4.62)	<0.001	Moderate	Outcome measurement bias existed in some studies; high heterogeneity (I²=81%) in the aerobic exercise subgroup of the subgroup analysis
Single-leg standing with eyes closed	7.03 (6.05, 8.02)	<0.001	Moderate	Outcome measurement bias existed in some studies; high heterogeneity (I²=81%) in the exercise cycle subgroup of the subgroup analysis
Grip strength	1.23 (0.06, 2.39)	0.04	Low	No statistically significant differences were found in all subgroups of the subgroup analysis; outcome measurement bias existed in some studies; the effect size was weak
Choice reaction time	-0.03 (-0.05, -0.01)	0.002	Moderate	Outcome measurement bias existed in some studies; moderate heterogeneity (I²=32%) in the exercise mode subgroup of the subgroup analysis
Vital capacity	86.81 (-1.56, 175.17)	0.05	Low	The overall effect was not statistically significant; publication bias existed (P<0.05 in Egger’s test); high heterogeneity (I²=56%) in subgroups

## Discussion

This study used Meta-Analysis to explore the impact of exercise intervention on the physical health of compulsory detoxification personnel. The results of the 18 included studies showed that, compared with the control group, exercise intervention led to statistically significant improvements in sit-and-reach, single-leg stance with eyes closed, and choice reaction time. While a positive trend was observed for grip strength [MD = 1.23, 95%CI (0.06, 2.39), P = 0.04], the finding for vital capacity [MD = 86.81, 95%CI (-1.56, 175.17), P = 0.05] did not achieve statistical significance. Furthermore, in the subgroup analysis, several comparisons related to kinematic indicators were not statistically significant.

While this meta-analysis provides valuable insights, several limitations should be considered. First, the number of included studies for some outcome measures was relatively small, which may limit the generalizability of the findings. Second, and importantly, the extensive number of subgroup analyses conducted across multiple outcomes and moderators increases the risk of Type I errors (false-positive findings). As these subgroup analyses were primarily exploratory and hypothesis-generating, the results, particularly those with p-values near the 0.05 threshold, should be interpreted with caution.

The study samples were all sourced from China, with a lack of cross-regional and cross-cultural data, which limited the generalizability of the conclusions; Future research should focus on confirmatory trials that pre-specify a limited number of subgroup hypotheses based on the preliminary evidence provided by this meta-analysis and other mechanistic studies. This would help to validate the optimal exercise prescription parameters identified here.

Based on the principles of sports training science and the conventional assessment principles of exercise effects, the content, intensity, and duration (total amount) of exercise are the main factors affecting the effect of exercise intervention (34). In the subgroup analysis, the differences in the intervention effects of two different types of exercise—aerobic exercise and traditional fitness exercise—on the physical health of compulsory detoxification personnel were compared. The results showed that aerobic exercise had significantly better improvement effects than traditional fitness exercise on sit-and-reach (MD = 4.07) and choice reaction time (MD=-0.04).This may be attributed to the fact that the continuous rhythmic movements of aerobic exercise promote improvements in muscle connective tissue elasticity and joint mobility (34);thereby enhancing flexibility. Regarding choice reaction time, the advantage of aerobic exercise is more likely explained by neural mechanisms—such as improved central processing efficiency, faster sensorimotor transmission, and enhanced corticospinal excitability—rather than solely by cardiopulmonary improvements (9). However, traditional fitness exercise showed better effects than aerobic exercise on single-leg stance with eyes closed (MD = 5.49) and vital capacity (MD = 131.47).This is because traditional exercise emphasizes “the unity of body and mind,” and its slow and controllable movement patterns can strengthen the coordination between proprioception and the vestibular system, thereby improving balance ability (29). Moreover, its characteristic of “abdominal breathing” can directly enhance the strength of the diaphragm and respiratory muscles, thereby improving pulmonary ventilation efficiency (25). In the subgroup analysis, the type of exercise did not have a significant impact on grip strength. This finding aligns with the principle of training specificity from exercise physiology: strength gains are primarily driven by neural adaptations (e.g., increased motor unit recruitment, firing rate synchronization) and muscle hypertrophy, both of which require high-intensity resistance training involving mechanical tension and metabolic stress. Aerobic and traditional fitness exercises, which formed the majority of interventions in this review, typically involve low-to-moderate intensity and lack the high-threshold motor unit activation necessary for stimulating strength adaptations. Therefore, the absence of grip strength improvement does not imply muscle atrophy, but rather reflects the insufficient strength-specific stimulus provided by the exercise protocols. Future interventions aiming to improve grip strength should incorporate progressive resistance training (e.g., handgrip strengtheners, wrist curls) with intensities ≥60–70% of 1RM, in accordance with NSCA guidelines (38), to induce both neural and hypertrophic adaptations (35).

Exercise cycle is one of the most critical factors determining exercise effects. The research data and subgroup analysis show that exercise lasting ≤ 12 weeks has better intervention effects than exercise lasting > 12 weeks on the sit-and-reach, choice reaction time, and vital capacity of compulsory detoxification personnel. This may be attributed to the fact that flexibility and neural responses, such as reaction time, are more sensitive to short-term exercise stimuli, often showing rapid improvements due to enhanced neuromuscular coordination and muscle elasticity (10). In contrast, cardiopulmonary function, including vital capacity, typically requires longer training periods and progressive overload to achieve significant adaptation. According to the National Strength and Conditioning Association (NSCA), the respiratory system is not heavily stressed during most endurance exercises, and its chronic adaptations are relatively slow and modest unless training intensity and volume are systematically increased over time (38). Therefore, the lack of significant improvement in vital capacity in longer-duration trials may be due to the absence of progressive overload, leading to physiological stagnation once the body adapts to a fixed workload. However, the exercise intervention lasting > 12 weeks has a better impact on the single-leg stance with eyes closed. This is because balance ability relies on the long-term remodeling of neuromuscular pathways. Long-term traditional fitness exercise can repeatedly strengthen motor memory, promote the coordinated regulation between the cerebellum and the somatic motor cortex, thereby achieving a gradual improvement in balance ability (33). Grip strength showed no significant improvement across different intervention periods (MD = 1.16 for ≤12 weeks, MD = 0.46 for >12 weeks). This does not necessarily indicate “irreversible” muscle or nerve damage, but rather reflects the lack of strength-specific training stimuli in the included interventions. According to NSCA (38) strength training principles, improvements in maximal strength require progressive overload with intensities sufficient to recruit high-threshold motor units (Type II fibers) and induce neuromuscular adaptations. Since most exercise protocols in this review were aerobic or traditional fitness-based, they failed to provide the necessary mechanical tension and metabolic stress for grip strength gains. Therefore, future interventions should integrate targeted resistance training (e.g., grip-specific exercises with progressive loads) combined with adequate protein intake and sufficient recovery, while extending the intervention period to allow for both neural and structural adaptations (36).

Exercising ≤ 3 days per week showed a better intervention effect on the single-leg stance with eyes closed in compulsory detoxification personnel than exercising > 3 days per week. Low-frequency training can reduce excessive fatigue of the vestibular system, which is more conducive to the accurate acquisition of balance control ability (21). However, exercising more than 3 days a week has a better intervention effect on choice reaction time and sit-and-reach than exercising ≤ 3 days a week. High-frequency stimulation can maintain muscle elasticity and nerve sensitivity, avoiding “adaptation fading caused by excessively long training intervals” (34). Improvements in vital capacity and grip strength require supplementary resistance training (such as dumbbell grip lifts) and breathing training (such as abdominal breathing) to make up for the limitations of a single type of exercise (35).

From the perspective of intervention duration, interventions with a duration of ≤ 40 minutes showed better effects on single-leg stance with eyes closed and vital capacity than those with a duration of > 40 minutes. However, interventions with a duration of more than 40 minutes had better effects on sit-and-reach and choice reaction time of compulsory detoxification personnel than those with a duration of ≤ 40 minutes. This is because a longer duration can ensure sufficient muscle stretching and continuous activation of nerve pathways (7). In the subgroup analysis, the intervention effect of exercise duration (single session duration) on grip strength showed a special pattern:After dividing the single exercise duration into two groups: ≤40 minutes and >40 minutes, the effect sizes of both groups were not statistically significant. However, the data trend showed that the effect size value of the ≤40-minute group was higher, suggesting that shorter-duration exercise may be more conducive to the potential improvement of grip strength. This may be closely related to the long-term damage caused by drugs to muscles and the nervous system. Drugs (such as methamphetamine) can inhibit protein synthesis, accelerate muscle fiber decomposition, and damage motor nerve conduction pathways. As a result, the recovery of muscle strength requires a longer period of targeted training, rather than simply relying on the extension of exercise duration (6). Therefore, simply adjusting the duration of a single exercise session may be difficult to significantly improve grip strength. It is necessary to combine the progressive increase in the intensity of resistance training (such as gradually increasing the resistance of grip strengtheners), nutritional supplementation (such as increasing protein intake), and nerve repair interventions (such as physical factor therapy) to more effectively promote the recovery of grip strength (36).

It can also be found from the aggregated results of this meta-analysis that, across the included studies, there were no statistically significant differences between the experimental and control groups at baseline before the exercise interventions. However, following the interventions, a significant difference emerged between the groups, which reflects the overall effectiveness of exercise intervention as synthesized from the included RCTs. Although significant positive effects have been observed, more research is still needed to further explore the underlying mechanisms through which physical exercise impacts physical health (33). Meanwhile, future research should focus on optimizing training programs and exploring their long-term impacts (37). Therefore, when formulating physical health intervention programs for compulsory detoxification personnel, multiple factors such as intervention measures, cycles, intervention frequency, and duration should be considered. This is to ensure the accuracy and reliability of evaluation results, provide a scientific basis for formulating more precise exercise intervention programs, and promote the effective improvement of the physical health of compulsory detoxification personnel.

## Conclusion

The purpose of this study is to evaluate the effectiveness of exercise intervention on the physical health of compulsory detoxification personnel. According to the meta-analysis, exercise intervention can lead to significant improvements in specific physical health indicators. The findings demonstrate statistically significant enhancements in sit-and-reach (MD = 3.92, 95%CI [3.23, 4.62]), single-leg stance with eyes closed (MD = 7.03, 95%CI [6.05, 8.02]), and choice reaction time (MD=-0.03, 95%CI [-0.05, -0.01]). While a positive effect was observed for grip strength (MD = 1.23, 95%CI [0.06, 2.39], P = 0.04), the improvement in vital capacity (MD = 86.81, 95%CI [-1.56, 175.17], P = 0.05) was not statistically significant.

Based on this evidence-based medical evidence, it is recommended to incorporate aerobic exercise intervention methods into the improvement of sit-and-reach and choice reaction time in compulsory detoxification personnel; in conjunction with exercising more than 3 times a week, each session lasting more than 40 minutes, and continuing for ≤ 12 weeks. To improve the ability of single-leg stance with eyes closed, traditional fitness exercise is more suitable, with exercising ≤ 3 times a week, each session lasting ≤ 40 minutes, and continuing for > 12 weeks. For optimizing vital capacity and grip strength, traditional fitness exercise is recommended, with exercising ≤ 3 times a week, each session lasting ≤ 40 minutes, and continuing for ≤ 12 weeks.

In conclusion, this study confirms the effectiveness of exercise intervention in improving the physical health of compulsory detoxification personnel. It also provides strong evidence for formulating more scientific and reasonable exercise intervention plans. For drug rehabilitation workers, compulsory detoxification personnel, and policy makers, these findings will contribute to the improvement and implementation of physical health initiatives for compulsory detoxification personnel, and better provide an evidence-based basis for enhancing their physical health levels.

## Data Availability

The original contributions presented in the study are included in the article/supplementary material. Further inquiries can be directed to the corresponding author.

## Glossary

RCTs: Randomized Controlled Trials
MD: Median difference
CI: Confidence interval
BMI: Body Mass Index
MeSH: Medical Subject Headings
PRISMA: Preferred Reporting Items for Systematic Reviews and Meta-Analyses
CNKI: China National Knowledge Infrastructure
T: experimental group
C: control group
HIIT: High Intensity Interval Training
%HRmax: percentage of maximum heart rate
%1RM: percentage of one-repetition maximum
RPE: Rating of Perceived Exertion, 6–20 scale

## References

[1] Ye X Liu R . Intervention effect of aerobic exercise on physical fitness, emotional state and mental health of drug addicts: a systematic review and meta-analysis. Int J Environ Res Public Health. (2023) 20:2272. doi: 10.3390/ijerph20032272. PMID: 36767656 PMC9916365

[2] Li H Wang C Huang X Xu L Cao Y Luo J . Chan-Chuang and resistance exercise for drug rehabilitation: a randomized controlled trial among Chinese male methamphetamine users. Front Public Health. (2023) 11:1180503. doi: 10.3389/fpubh.2023.1180503. PMID: 37965508 PMC10642185

[3] Jia D Xu Y . Effects of an 8-week Baduanjin intervention combined with low-carbohydrates diet among overweight people who struggle with drug addiction. Front Public Health. (2022) 10:989519. doi: 10.3389/fpubh.2022.989519. PMID: 36339240 PMC9633992

[4] Jia D Zhou J Xu Y . Effectiveness of traditional Chinese health-promoting exercise as an adjunct therapy for drug use disorders: a systematic review and meta-analysis. J Integr Complement Med. (2022) 28:294–308. doi: 10.37766/inplasy2021.5.0066. PMID: 35426734

[5] Huang WX Zhang QY Li XD Tian YQ Huang J Zhang R . Research progress on biomarkers of drug addiction. Chin J Comp Med. (2025) 35:147–57. doi: 10.3969/j.issn.1671-7856.2025.02.016

[6] Zhang XL Zhan SQ Zhang L Qian SB . Physical characteristics of adult males in compulsory isolation drug rehabilitation. Mod Sports Sci Technol. (2018) 8:242–5. doi: 10.16655/j.cnki.2095-2813.2018.01.242

[7] Gu Q Sheng L Ma XM . Research progress on exercise-based drug rehabilitation methods and effects for drug addicts. Sports Sci. (2019) 40:37–45. doi: 10.13598/j.issn1004-4590.2019.06.006

[8] Huang J Zheng Y Gao D Hu M Yuan T . Effects of exercise on depression, anxiety, cognitive control, craving, physical fitness and quality of life in methamphetamine-dependent patients. Front Psychiatry. (2020) 10:999. doi: 10.3389/fpsyt.2019.00999. PMID: 32047445 PMC6997340

[9] Wang DS Zhu T . Effects of aerobic exercise on physical fitness, craving, and emotional state in methamphetamine-dependent individuals. China Sports Sci. (2017) 37:50–9. doi: 10.16469/j.css.201707007

[10] Tian JF Yang J . Systematic review of health and functional benefits of aerobic exercise rehabilitation for mental and behavioral disorders caused by substance abuse. Chin J Rehabil Theory Pract. (2023) 29:443–51. doi: 10.3969/j.issn.1006⁃9771.2023.04.010

[11] Page MJ McKenzie JE Bossuyt PM Boutron I Hoffmann TC Mulrow CD . The PRISMA 2020 statement: an updated guideline for reporting systematic reviews. BMJ. (2021) 372:n71. doi: 10.31222/osf.io/v7gm2. PMID: 33782057 PMC8005924

[12] Luo Y Zhang S Shang H Cui W Wang Q Zhu B . Prevalence of Clostridium difficile infection in the hematopoietic transplantation setting: update of systematic review and meta-analysis. Front Cell Infect Microbiol. (2022) 12:801475. doi: 10.3389/fcimb.2022.801475. PMID: 35265530 PMC8900492

[13] Sun J Sun Z Kong J Tian X Wang L Wang Q . Regular meta-analysis of the impact of sports activities intervention on some items of the national student physical health standards for adolescents. Front Physiol. (2024) 15:1419441. doi: 10.3389/fphys.2024.1419441. PMID: 39512469 PMC11540668

[14] Higgins JPT Altman DG Gøtzsche PC Jüni P Moher D Oxman AD . The Cochrane Collaboration's tool for assessing risk of bias in randomised trials. BMJ. (2011) 343:d5928. doi: 10.1136/bmj.d5928. PMID: 22008217 PMC3196245

[15] Wang HP Yuan CF Zhang JL Li QY . Meta-analysis of the effect of traditional Chinese medicine exercise therapy on mental behavior, physical health, and quality of life in drug addicts. China Sport Sci Technol. (2021) 57:67–76. doi: 10.1249/01.mss.0000517680.68840.5f. PMID: 41871379

[16] Liu ZQ Du XD Zhang AM Lin JX Gong YZ . Effects of motor function training on physical fitness of compulsory rehabilitation personnel. Sports Sci Res. (2021) 25:48–53. doi: 10.1007/978-1-4419-9863-7_455. PMID: 41811545

[17] Liu YZC Han YM Meng LS Lu H Dong J Lu H . Beneficial effects of 16-week high-intensity interval training on physical health of compulsory isolation drug rehabilitation personnel. China Sport Sci Technol. (2021) 57:31–7. doi: 10.1016/j.cger.2019.07.011. PMID: 31543179 PMC6760312

[18] Yuan RQ Huang HF Liu DD Yu H Liang LY Feng GR . Study on physical fitness rehabilitation effect of progressive exercise training for drug addicts. Chin J Drug Abuse Prev Treat. (2018) 24:200–4. doi: 10.15900/j.cnki.zylf1995.2018.04.004

[19] Gui Z Luo L Yuan DZ Cheng QZ Tang Y . Effects of health education combined with long-term aerobic exercise on psychology, sleep, and physical fitness in elderly male depressed drug addicts. Mod Prev Med. (2021) 48:1859–62. doi: 10.20043/j.cnki.mpm.2021.10.031

[20] Wu JL Jiang XP Deng YW Fa SY . Study on physical fitness rehabilitation of compulsory isolated drug addicts through different exercise interventions. J Yunnan Police Coll. (2021) 1:106–9.

[21] Yang J . Effects of 12-week HIIT on immune cell response and balance ability of male compulsory drug rehabilitation personnel. Bull Sport Sci Technol. (2024) 32:280–2. doi: 10.19379/j.cnki.issn.1005-0256.2024.08.068

[22] Yang XG Zhou XX Song E Xiang YY Liu JR Zhang JY . Application of aerobic exercise prescription in health management of drug rehabilitation personnel. Health Exam Manag. (2024) 5:178–81.

[23] Ma Q Wen J Zheng TL . Effects of VR exercise intervention on physical fitness rehabilitation of drug addicts. J Appl Technol. (2023) 23:391–5. doi: 10.3969/j.issn.2096-3424.2023.04.014

[24] Han QH Fang CL . Experimental study on the effects of gas volleyball on physical fitness of heroin-addicted rehabilitation personnel. Mod Sports Sci Technol. (2020) 10:230–3. doi: 10.16655/j.cnki.2095-2813.2020.15.230

[25] Wang SF Zhang LQ Wang L . Study on physical fitness intervention of traditional health-preserving sports combination exercise on HIV/AIDS compulsory rehabilitation population. J Yuxi Norm Univ. (2019) 35:93–8.

[26] Li SY . Effects of aerobic exercise combined with resistance training on physical and mental rehabilitation of methamphetamine addicts. Chin J Drug Depend. (2022) 31:188–93. doi: 10.13936/j.cnki.cjdd1992.2022.03.006

[27] You YH Song X Wen JF Wu M Wang Q Kang YZ . Effects of "dance-body combined training" on physical health of female compulsory drug rehabilitation personnel. Sports Sci Technol. (2022) 43:45–7. doi: 10.14038/j.cnki.tykj.2022.06.020

[28] Fischetti F Cataldi S Di Terlizzi P Greco G . Multilateral methodology in physical education improves coping skills, resilience and physical fitness in drug addicts. J Hum Sport Exerc. (2020) 15(2):367–79. doi: 10.14198/jhse.2020.152.11

[29] Zhang Z Zhu D . Effect of Taijiquan exercise on rehabilitation of male amphetamine-type addicts. Evid Based Complement Alternat Med. (2020) 2020:1–9. doi: 10.1155/2020/8886562. PMID: 33293997 PMC7690993

[30] Zhu D Jiang M Xu D Schöllhorn WI . Long-term effects of mind-body exercises on the physical fitness and quality of life of individuals with substance use disorder—a randomized trial. Front Psychiatry. (2020) 11:528373. doi: 10.3389/fpsyt.2020.528373. PMID: 33391039 PMC7775308

[31] Menglu S Ruiwen L Suyong Y Dong Z . Effects of Tai Chi on the executive function and physical fitness of female methamphetamine dependents: a randomized controlled trial. Front Psychiatry. (2021) 12:653229. doi: 10.3389/fpsyt.2021.653229. PMID: 34177646 PMC8222617

[32] Xu J Zhu Z Liang X Huang Q Zheng T Li X . Effects of moderate-intensity exercise on social health and physical and mental health of methamphetamine-dependent individuals: a randomized controlled trial. Front Psychiatry. (2022) 13:997960. doi: 10.3389/fpsyt.2022.997960. PMID: 36213929 PMC9539410

[33] Zhang L Zeng H Sun Y Xue H Gao L Zhu W . Effect of Tai Chi compared to running on drug cravings, attention bias, and physical fitness in men with methamphetamine use disorder. Healthcare. (2024) 12:1653. doi: 10.3390/healthcare12161653. PMID: 39201211 PMC11353623

[34] Bellón JÁ Conejo-Cerón S Sánchez-Calderón A Rodríguez-Martín B Bellón D Rodríguez-Sánchez E . Effectiveness of exercise-based interventions in reducing depressive symptoms in people without clinical depression: systematic review and meta-analysis of randomised controlled trials. Br J Psychiatry. (2021) 219:578–87. doi: 10.1192/bjp.2021.105, PMID: 33533706

[35] Dou HB Cheng BJ He H . Study on the effects of circuit resistance training on muscle strength and aerobic endurance of female college students. J Guangzhou Sport Univ. (2020) 40:87–90. doi: 10.13830/j.cnki.cn44-1129/g8.2020.02.021

[36] Wang ZZ . Research and application progress of exercise prescription. Sports Sci Res. (2021) 35:40–9. doi: 10.15877/j.cnki.nsic.20210601.001

[37] Zhang X Li Y Zhao M . Collective exercise therapy on quality of life and emotions in female new drug addicts. Chin J Drug Rehabil. (2024) 23:189–95.

[38] Haff GG Triplett NT . NSCA's essentials of personal training. 2nd ed. Champaign, IL: Human Kinetics (2021).

